# Single-cell profiling identifies a pro-tumoral VCAN positive macrophage subset and defines a prognostic signature in glioblastoma

**DOI:** 10.1007/s12672-026-05166-y

**Published:** 2026-05-09

**Authors:** Jiaxin Guo, Zhansheng Zhu, Nanyang Tong, Dingding Xu, Huan Zhang, Chenshi Lin, Guiping Wan, Yamei Wang, Qingqing Zhou, Liang Xia

**Affiliations:** 1 Department of Neurology, The First Affiliated Hospital of Yangtze University, Jingzhou First People’s Hospital, Jingzhou, 434000 China; 2 Graduate student, Yangtze University, Jingzhou, 434000 China; 3 Department of Neurosurgery, The First Affiliated Hospital of Yangtze University, Jingzhou First People’s Hospital, Jingzhou, 434000 China; 4 Postgraduate training base Alliance of Wenzhou Medical University, Wenzhou, 325035 Zhejiang China; 5 Department of Neurosurgery, Zhejiang Cancer Hospital, Hangzhou Institute of Medicine (HIM), Chinese Academy of Sciences, Hangzhou, 310022 Zhejiang China; 6 Department of Oncology, The First Affiliated Hospital of Yangtze University, Jingzhou First People’s Hospital, Jingzhou, 434000 China

**Keywords:** Glioblastoma, Macrophage, VCAN⁺ macrophage subpopulation, Single-cell RNA sequencing, Immune microenvironment

## Abstract

Glioblastoma (GBM), the most aggressive primary brain tumor, develops within a tumor microenvironment (TME) dominated by tumor-associated macrophages (TAMs) that critically influence disease progression. Through single-cell RNA sequencing (scRNA-seq) and bioinformatic analysis, we delineated macrophage heterogeneity within the GBM TME and identified a distinct VCAN⁺ macrophage subpopulation. Pseudotime trajectory analysis revealed these cells at the terminal stage of macrophage differentiation, where they exhibit enhanced granulocyte migration and chemotaxis pathways and exhibit a pro-tumorigenic phenotype that diverges from classical M1/M2 polarization. These VCAN⁺ macrophages displayed a distinct polarization state driven by tumor necrosis factor-α (TNF-α), contributing to both a pro-inflammatory and an immunosuppressive TME. CellChat analysis demonstrated their pivotal role in intercellular communication—predominantly mediating crosstalk with GBM tumor cells, endothelial cells, and CD8⁺ T cells via *SPP1* signaling: *SPP1* binding to *CD44* on tumor cells enhances their invasiveness, while its interaction with *CD47* on CD8⁺ T cells inhibits anti-tumor immunity. Transcriptional regulatory network analysis identified that *CDX2* and *MXI1* serve as key transcription factors modulating VCAN⁺ macrophage function and maintaining their specific polarization and homeostasis. Through machine learning, we identified seven hub genes—*C1QA*,* C1QC*,* C3*,* CCL4*,* CD44*,* SERPINE1*, and *TREM2* (all highly expressed in VCAN⁺ macrophages and involved in their polarization or intercellular communication)—and constructed an effective GBM prognostic model (AUC = 0.83 in validation cohort), underscoring their roles in immune regulation, extracellular matrix remodeling, and VCAN⁺ macrophage-mediated tumor progression. Further studies with larger cohorts and functional validation will clarify this subpopulation’s therapeutic potential and spatial distribution patterns.

**Supplementary Information:**

The online version contains supplementary material available at 10.1007/s12672-026-05166-y.

## Introduction

Glioblastoma (GBM) is the most prevalent and aggressive primary brain tumor, accounting for ~ 14–15% of all malignant primary brain tumors. Epidemiological data indicate that the occurrence rate of GBM increases with age, with a median age at diagnosis of approximately 64 years, and the incidence is significantly higher in males than in females [1]. In Pakistan, studies have shown that the mean age at diagnosis for GBM patients is 45 years, with males comprising 68.8% of cases [2]. The tumors primarily occur in the cerebral hemispheres, particularly in the frontal, temporal, and parietal lobes. The clinical presentation varies depending on anatomical location, typically manifesting as headaches, cognitive dysfunction, seizures, or focal neurological deficits. The diagnosis of GBM relies mainly on magnetic resonance imaging (MRI) combined with histopathological examination [3]. The standard treatment for GBM combines maximal safe resection, radiotherapy, and temozolomide-based chemotherapy [4]. Even with this aggressive multimodal approach, outcomes remain dismal, with median survival limited to 14–16 months [5]. Tumor recurrence occurs frequently, and fewer than 10% of patients survive beyond five years [4]. Studies have demonstrated that age, ethnicity, tumor location and size, as well as treatment modalities, are independent prognostic factors [5]. Notably, patients with early-detected and diagnosed GBM (incidental GBM) exhibit relatively better outcomes [6]. These observations highlight the necessity of deeper investigation into GBM pathophysiology—particularly the tumor microenvironment (TME), which drives treatment resistance and recurrence—and the discovery of new therapeutic targets to enhance outcomes and survival rates.

A key contributor to GBM’s dismal prognosis is the TME’s immune component, in which tumor-associated macrophages (TAMs) play a central role in promoting tumor progression and evading therapy. The tumor microenvironment (TME) comprises tumor cells, stromal cells, immune cells, and extracellular matrix, collectively forming a critical ecosystem that governs tumor progression. The microenvironment of GBM is particularly complex, in which immune cells are key regulators of tumor progression. Tumor-associated macrophages (TAMs) are the most abundant immune cell type in the GBM microenvironment, accounting for approximately 30% of the tumor cell population and significantly contributing to both tumor initiation and progression [7]. TAMs originate primarily from tissue-resident microglia and bone marrow-derived macrophages (MDMs), contributing critically to glioblastoma progression, therapeutic resistance, and immune evasion [8]. Macrophages are highly plastic and capable of polarizing into either M1-type (anti-tumor) or M2-type (pro-tumor) phenotypes. Single-cell RNA sequencing (scRNA-seq) has uncovered substantial heterogeneity and dynamic evolution in TAMs, distinguishing up to seven distinct subsets that participate in GBM immune regulation through diverse ligand-receptor interactions and signaling pathways [9]. Notably, GBM cell-mediated reprogramming predominantly polarizes TAMs toward the M2 phenotype, thereby promoting tumor growth, invasion, angiogenesis, immunosuppression, and treatment tolerance [10]. M2-type TAMs secrete pro-inflammatory cytokines, establishing a positive feedback loop via pathways such as the IFNγ-IRF2-ARPC1B axis that enhances tumor cell migration, invasion, and epithelial-mesenchymal transition [11]. Therefore, targeted modulation of TAM polarization (e.g., promoting the conversion of M2-type to M1-type) has become an important strategy for GBM immunotherapy, as it may reshape the TME and potentiate antitumor immunity [12]. This intricate interplay underscores the urgent need to dissect macrophage heterogeneity and functional states for developing precision immunotherapies.

Recent advances in scRNA-seq have revolutionized our ability to characterize tumor heterogeneity. This approach facilitates high-throughput gene expression profiling at single-cell resolution, with exceptional precision revealing the intricate cellular composition of tumor microenvironments [13]. In GBM research, scRNA-seq has uncovered substantial intra-tumoral heterogeneity, identifying distinct malignant cell subpopulations while delineating immune cell profiles and their spatial organization [14]. This technology has enabled researchers to identify several functionally distinct cellular subpopulations, including mesenchymal-2-like (MES2-like) GBM cells—which exhibit dual glial-immune properties (expressing GFAP, a glial marker, and IL-6, an immune regulatory cytokine)—and are strongly associated with immune evasion and unfavorable clinical outcomes [15]. In addition, scRNA-seq has revealed critical signaling networks in the tumor microenvironment, including CXCL, EGF, FGF, and MIF pathways, which significantly influence tumor progression [14]. In clinical practice, molecular markers derived from scRNA-seq data have facilitated the construction of prognostic models for GBM patients, providing novel avenues for personalized treatment strategies [16]. Integrating single-cell and spatial transcriptomics has revealed the spatial distribution of distinct GBM subpopulations and their metabolic diversity, offering new perspectives for developing targeted therapies [17]. While single-cell sequencing has revolutionized our understanding of GBM TME heterogeneity, the functional mechanisms of specific macrophage subpopulations (e.g., those with unique molecular signatures) and their contributions to tumor progression remain incompletely characterized—creating a critical gap in developing targeted immunotherapies.

This study systematically identified key immune cell subpopulations in the GBM microenvironment, particularly macrophages, through scRNA-seq and bioinformatics analysis, and investigated their functional mechanisms in tumor progression. The innovation of the study is to deeply analyze the molecular characteristics of the VCAN⁺ macrophage subpopulation and its functional significance in GBM development, and this specific subpopulation may play a unique and critical role in the GBM microenvironment. By combining single-cell and conventional transcriptomics data with complementary bioinformatics approaches—including CellChat analysis, pseudotime trajectory analysis, and transcriptional regulatory network analysis—we characterized the VCAN⁺ macrophage subpopulation and elucidated its dynamic interactions with tumor cells. Clinically, identifying these key immune cell subpopulations and their markers holds substantial importance for GBM patients, as it could facilitate the discovery of novel therapeutic targets and prognostic biomarkers. This approach may also advance the development of immunotherapeutic strategies for GBM, potentially improving treatment outcomes and patient survival for this aggressive malignancy.

## Materials and methods

### Data acquisition and preprocessing

The Gene Expression Omnibus (GEO) database (https://www.ncbi.nlm.nih.gov/geo/*)* provides extensive scRNA-seq data. In this study, we obtained the scRNA-seq dataset GSE162631 from the GEO database, which includes tumor core samples from four GBM patients and matched peripheral tissue samples from the same individuals. The dataset was generated using magnetically-activated cell sorting to enrich CD31⁺ GBM endothelial cells. In this study, the GSE162631 dataset was imported and processed using the Seurat package (version 4.2.0) [18] in R. Low-quality cells and genes were filtered by the following criteria. Firstly, Cells with no detectable gene expression were removed. Secondly, Cells expressing between 200 and 7,500 genes were retained. Thirdly, Cells with fewer than 75,000 unique molecular identifier (UMI) counts were excluded. Finally, Cells with a percentage of mitochondrial genes less than 10% were retained. The data were normalized using the “Normalize Data” function in the Seurat R package. Highly variable genes were subsequently identified by balancing their average expression against dispersion. Principal component analysis (PCA) was subsequently performed, and significant principal components (PCs) were used as input for graph-based clustering. To mitigate batch effects across samples, we applied the harmony integration method. Cell clusters were identified using the FindClusters function, which implements a shared nearest neighbor (SNN) modularity optimization-based algorithm, resulting in 35 distinct clusters derived from 37 principal components at a resolution of 1.4. Uniform manifold approximation and projection (UMAP) was subsequently performed using the “Run UMAP” function. Cell aggregation patterns were visualized using the UMAP-1 and UMAP-2 coordinates. To identify differentially expressed genes between cell populations, we analyzed the normalized gene expression data using the Find All Markers function in Seurat with default parameters. Cell clusters were then annotated based on established cell type-specific biomarkers (Table S1) [19], and their respective proportions were quantified and assessed.

### Download and processing of bulk transcriptome data

The Chinese Glioma Genome Atlas (CGGA, http://www.cgga.org.cn/index.jsp*)* provides clinical and molecular sequencing data from over 2,000 brain tumor samples within a Chinese patient cohort [20]. We retrieved mRNA sequencing data (batch1 and batch2) for glioma from this resource, along with corresponding clinical information, which comprised a total of 1,018 samples. From this cohort, we selected 374 WHO grade IV samples based on clinical annotations and used their expression matrices and survival information for prognostic modeling. Genome-wide expression profiling data and clinical data for gliomas (TCGA-GBM) in TPM format [21] were downloaded from The Cancer Genome Atlas (TCGA, https://portal.gdc.cancer.gov/*)* via the R package TCG Abiolinks (version 2.25.0). The TCGA-GBM cohort (*n* = 176) comprises 171 GBM tumor samples and five paired normal controls; 160 tumor samples with associated survival data served as the validation set for prognostic modeling. We also acquired glioma single nucleotide variation (SNV) data from the TCGA database, which were generated via the “VarScan2 Variant Aggregation and Masking” tool.

The CGGA and TCGA databases offer extensive data on cancer tissue samples; however, their focus on cancer types results in a limited number of normal tissue samples. In contrast, the Genotype-Tissue Expression (GTEx) database contains extensive data from normal tissue samples across numerous organs and tissues. To compare gene expression between gliomas and normal tissues, we obtained an integrated expression matrix from the TCGA and GTEx projects via the UCSC Xena database (https://xenabrowser.net/datapages/*)*, which comprised 166 glioma samples and 1,137 normal brain tissue samples. The expression matrices for 1,303 samples from the UCSC Xena database were merged with those of 374 WHO grade IV glioma samples obtained from the CGGA database. After removing 32 outlier samples, we corrected for batch effects using the normalize Between Arrays function from the R package limma (version 3.50.0) [22], yielding a final dataset of 508 glioma and 1,137 normal brain tissue samples.

### Cell type deconvolution

To map cell type information from single-cell data onto bulk transcriptome profiles, we applied the single-sample Gene Set Enrichment Analysis (ssGSEA) method. This method quantifies the expression level of a gene set within a single sample by calculating an enrichment score based on the cumulative expression of all genes in the set [23]. In this study, we implemented ssGSEA to bulk transcriptome data from 508 glioma and 1,137 normal brain tissue samples, using the top 100 significantly differentially expressed genes per cell type as signature gene sets to compute cell type-specific enrichment scores for each sample. Differences in cellular composition between the glioma and normal samples were assessed by comparing the scores of each cell type. Subsequently, correlation coefficients and their corresponding p-values were calculated for the cell types, and correlation heat maps were generated using the R package gcorrplot (version 0.1.4.999).

### Screening of key immune cells

To identify immune cells critically involved in glioma pathogenesis, we applied three complementary machine learning algorithms—support vector machine recursive feature elimination (SVM-RFE), least absolute shrinkage and selection operator (LASSO) regression, and random forest—to immune cell abundance data from glioma samples. Each algorithm independently assessed the importance of immune cell subtypes. SVM-RFE was employed to select key features by iteratively training the model and removing the least important immune cell subtypes at each step. LASSO regression identified associated subtypes by applying L1 regularization, which shrinks coefficients of less relevant features to zero through tuning of the parameter λ. The random forest algorithm evaluated importance by constructing multiple decision trees and using its intrinsic metric to rank subtypes by their contribution to predictive performance. To ensure robust and consensus results, we took the intersection of key subtypes identified by all three algorithms. These overlapping immune cell subtypes were considered high-confidence candidates associated with the disease state and were selected for further investigation.

To ensure the objectivity and robustness of feature selection, distinct cutoff strategies were applied for each machine learning algorithm based on their inherent mathematical principles and cross-validation results: SVM-RFE selected the gene subset corresponding to the minimum error via 10-fold cross-validation; LASSO regression identified the optimal model with the minimum λ value and an error within one standard deviation through cross-validation; the random forest algorithm retained features with an importance score > 0 by default (based on mean decrease in accuracy and mean decrease in Gini). No additional subjective cutoffs were introduced during the intersection analysis of the three algorithm results, and the overlapping immune cell subtypes were defined as high-confidence key features to reduce the contingency of a single method.

### Immune response enrichment analysis

Immune Response Enrichment Analysis (IREA), introduced by Ang Cui et al., infers cytokine activity and immune cell polarization from gene expression data in immunological studies [24]. This method identifies over 66 distinct cytokine-driven polarization states in immune cells, including several previously uncharacterized states, from a cell type-centric perspective. We employed IREA to infer cytokines predominantly produced by key immune cells and to determine the polarization status of these cells in glioma, based on differential gene expression between glioma and normal controls in the single-cell transcriptome dataset GSE162631 (log2Fold Change > 0.5, *p* < 0.05).

### Constructing cellular trajectories by pseudotime analysis

Cellular trajectories were reconstructed using pseudotime analysis in Monocle 2 (version 3.3.5) [25]. This method orders cells along a pseudotime axis based on a user-defined set of highly variable genes, using a reversed graph embedding approach to model both linear and branched differentiation processes. Prior to trajectory inference, raw count data from β cells were normalized by estimating size factors. Trajectories were constructed using genes exhibiting high dispersion (dispersion estimate ≥ 1) and adequate expression levels (average expression ≥ 0.1) [26]. The DDRTree algorithm was applied with its default parameters. To investigate lineage branching events, we performed Branch Expression Analysis Modeling (BEAM) [25], which identifies genes with significant branch-dependent expression patterns. The results of the BEAM analysis were visualized using heatmaps generated by Monocle 2.

### Intercellular communication analysis

Intercellular communication was inferred using the R package CellChat (v1.1.3) [27], which evaluates ligand-receptor interactions and uncovers signaling pathways between distinct cell types [28]. We applied this tool to the single-cell dataset GSE162631 to map communication networks between all cell types in GBM samples. Our analysis characterized afferent and efferent signaling patterns, quantified communication probabilities, and assessed interaction strengths. We focused on interactions involving key immune cells and selected the most significant signaling pathways for detailed visualization. For all analyses, the default significance threshold was set at a Benjamini-Hochberg (BH) corrected p-value ≤ 0.05.

### Single-cell regulatory network analysis

To identify key transcription factors (TFs) across distinct cell types, we conducted single-cell cis-regulatory network inference using pySCENIC (version 0.12.1) [29]. This method first reconstructs regulatory networks from gene co-expression patterns and subsequently identifies direct regulatory interactions—such as TFs and their target genes—via DNA motif enrichment analysis. Finally, we quantified the activity of these regulatory networks within individual cells by computing AUCell scores. This approach enabled assessment of cell-type-specific TF activities.

### Screening and enrichment analysis of key differentially expressed genes (DEGs)

Differentially expressed genes (DEGs) were identified by comparing glioma samples (*n* = 508) with normal controls (*n* = 1137) using bulk transcriptome sequencing data and the limma R package. A significance threshold of |log2Fold Change| > 1 and p-value < 0.05 was applied. The resulting DEGs were visualized via a volcano plot (ggplot2, v3.3.6) and a heatmap based on Euclidean distance and hierarchical clustering (pheatmap, v1.0.12).

These bulk tissue DEGs were then intersected with those from the key VCAN⁺ macrophage subpopulation in single-cell data (GSE162631; |log2Fold Change| > 1.5, *p* < 0.05) to obtain a core set of conserved, key DEGs.

To elucidate the biological functions of these key DEGs, Gene Ontology (GO) and Kyoto Encyclopedia of Genes and Genomes (KEGG) pathway enrichment analyses were performed using clusterProfiler (v4.2.2) with a significance cutoff of *p* < 0.05. GO analysis covered Biological Process (BP), Molecular Function (MF), and Cellular Component (CC) domains [30], while KEGG analysis identified significantly enriched metabolic and signaling pathways [31].A significance cutoff of *p* < 0.05 (raw p-value, without multiple testing correction) was applied for both analyses. This parameter setting was based on the exploratory nature of the enrichment analysis, which aimed to comprehensively reveal the potential biological pathways involved in key DEGs and provide clues for subsequent mechanistic investigations. Given that enrichment analysis itself is a secondary test of gene sets, and we prioritized pathways with clear biological significance and high enrichment significance, the use of raw p-values without multiple testing correction is an acceptable common practice for exploratory research.

### Machine learning screening of hub genes

**Fig. 1:**
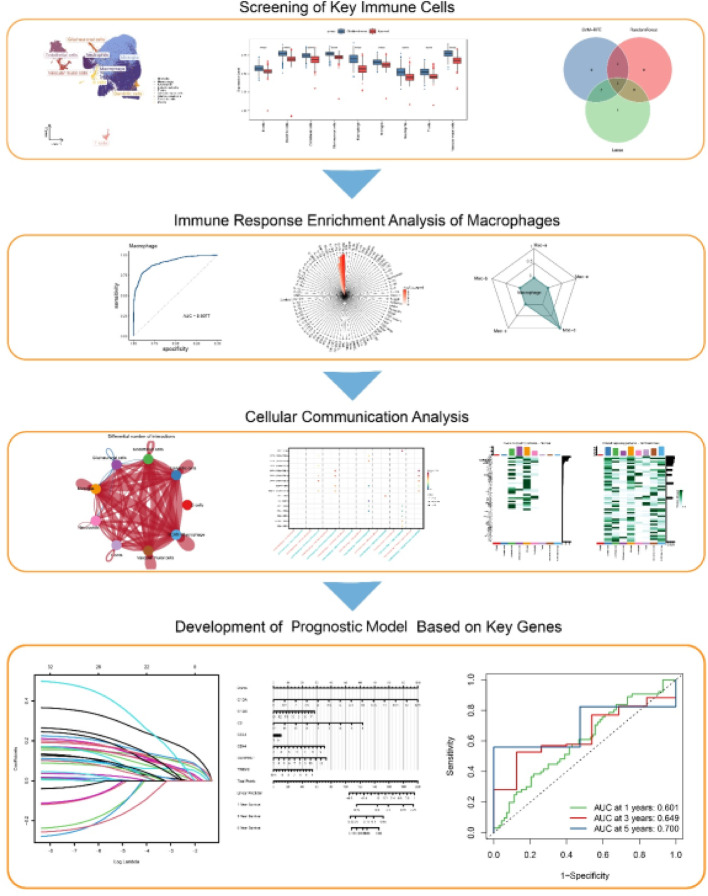
Illustrates the flow chart of this study

Hub genes were identified by integrating three feature selection algorithms. SVM-RFE was applied to recursively remove the least discriminative features. LASSO regression was performed using the R package glmnet (v2.25.0), which employs L1 regularization to penalize coefficients and retain informative features. For LASSO, features with a binomial distribution were used, and the model with the minimum error was selected. Random forest analysis was implemented using the randomForest package, with the mtry parameter tuned to minimize error and the ntree parameter set upon error stabilization. The top 10 most important features from the random forest model were selected based on both mean decrease in accuracy (MDA) and mean decrease in Gini (MDG). The intersection of feature genes identified by all three algorithms was taken to define a final set of high-confidence hub genes for subsequent analysis (see Fig. 1).

### Construction of the nomogram

Using the R package rms (version 6.3.0), we developed a nomogram based on the hub genes to visually predict 1-, 3-, and 5-year overall survival in patients. We subsequently assessed the nomogram’s performance using calibration curves and time-dependent ROC curves.

### Statistical analysis

All statistical analyses were conducted with R software (version 4.1.2). Kaplan–Meier survival analysis was performed using the “survival” and “survminer” packages. We evaluated correlations between two parameters via the Spearman correlation test and compared differences between two groups using the nonparametric Wilcoxon rank-sum test. A two-sided *P* < 0.05 indicated statistical significance.

## Results

### Single-cell dimensionality reduction, clustering, and annotation

**Fig. 2:**
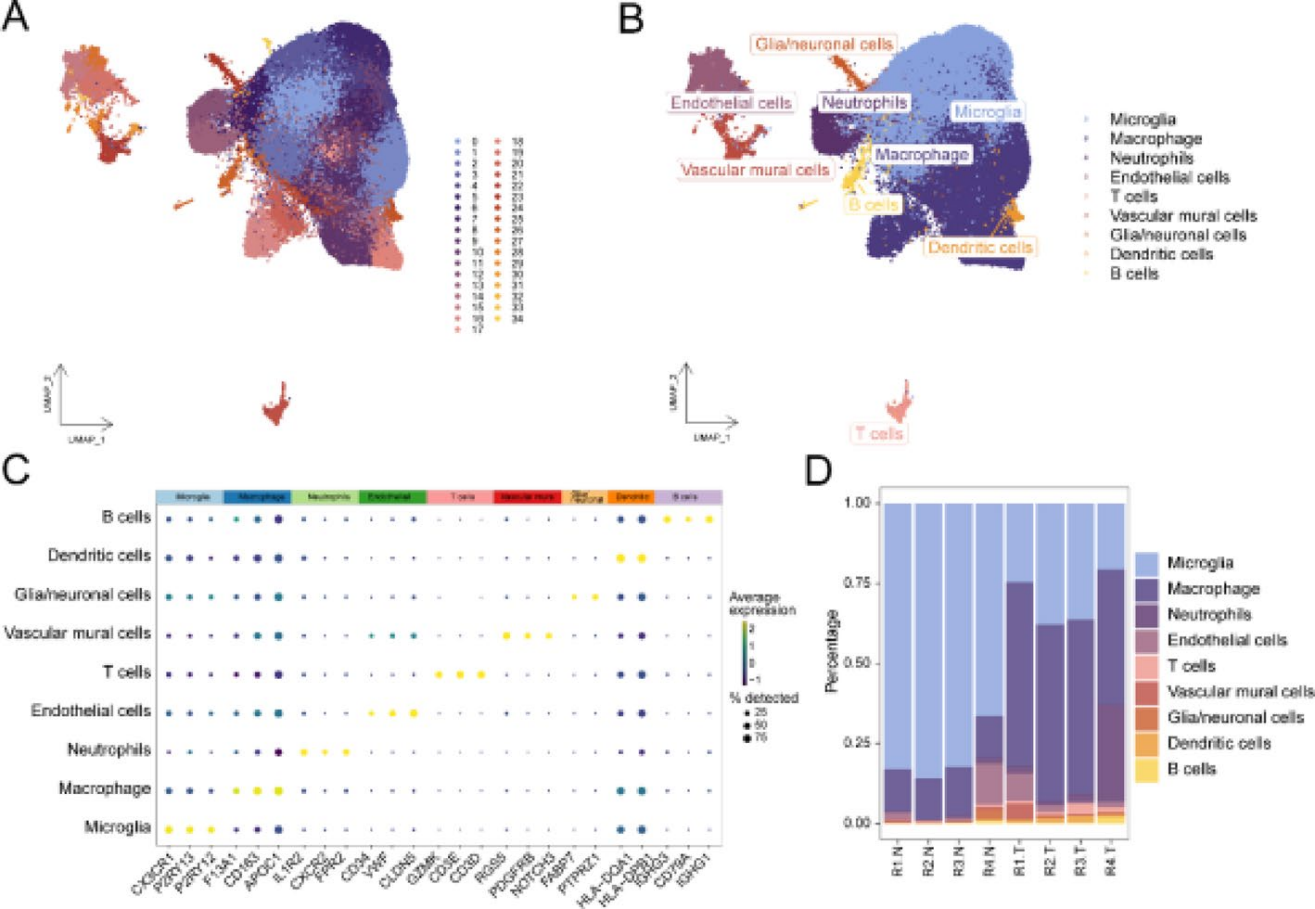
Identification of cellular heterogeneity in the GBM single-cell dataset GSE162631. **A** UMAP plot showing the 35 unsupervised clusters. **B** UMAP plot visualizing the nine annotated major cell types. **C** Dot plot of canonical marker gene expression used for cell type annotation. **(D)** Stacked bar chart showing the relative proportion of each cell type across four GBM tumor core samples (R1.T, R2.T, R3.T, R4.T) and four matched peripheral tissue samples (R1.N, R2.N, R3.N, R4.N)

We analyzed the GBM single-cell dataset GSE162631. Following initial quality control 97,377 high-quality cells were retained for subsequent analysis. These cells were clustered into 35 distinct groups (Fig. 2A); they were then annotated into specific cell types based on the expression of known marker genes (Fig. 2C, Table S1). We identified nine major cell types: microglia, macrophages, neutrophils, endothelial cells, T cells, vascular mural cells, glial/neuronal cells, dendritic cells, and B cells (Fig. 2B, Table S2). The proportional distribution of each cell type across individual samples is shown in Fig. 2D, which revealed a significantly higher abundance of macrophages within the GBM tumor core samples compared to peripheral tissues (Table S3).

### Cell type deconvolution

**Fig. 3:**
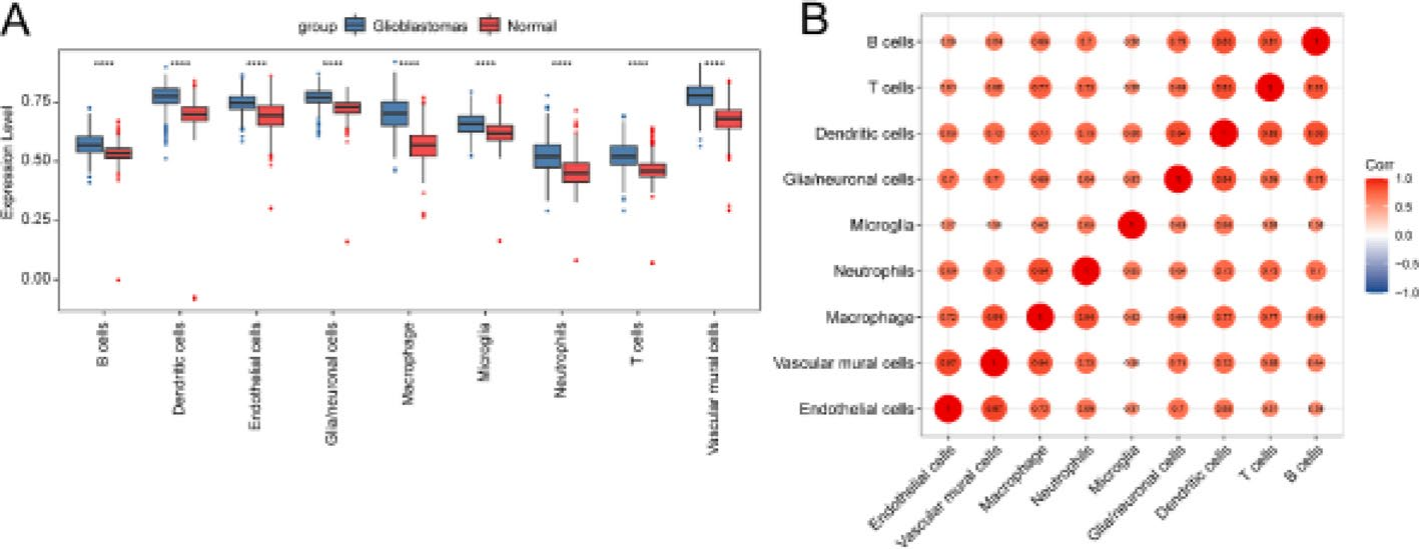
Differences in abundance of the nine cell types between groups and correlation analysis. **A** Box plot showing abundance differences of the nine cell types between GBM and normal samples. **B** Heatmap showing correlations among the nine cell types. Asterisks indicate p-values: ****p < 0.0001, ***p < 0.001, **p < 0.01, *p < 0.05

We deconvolved the abundances of nine cell types within bulk transcriptome sequencing samples (Table S5) using cell type specific DEGs (Table S4). This was done to quantify proportional changes in the tumor microenvironment following tumorigenesis; these proportions were subsequently used for machine learning. Compared to normal samples, GBM samples exhibited a significantly higher abundance across all nine cell types (*p* < 0.05, Fig. 3A). Correlation analysis revealed predominantly positive associations among these cell types (Fig. 3B).

### Screening of key immune cells

**Fig. 4:**
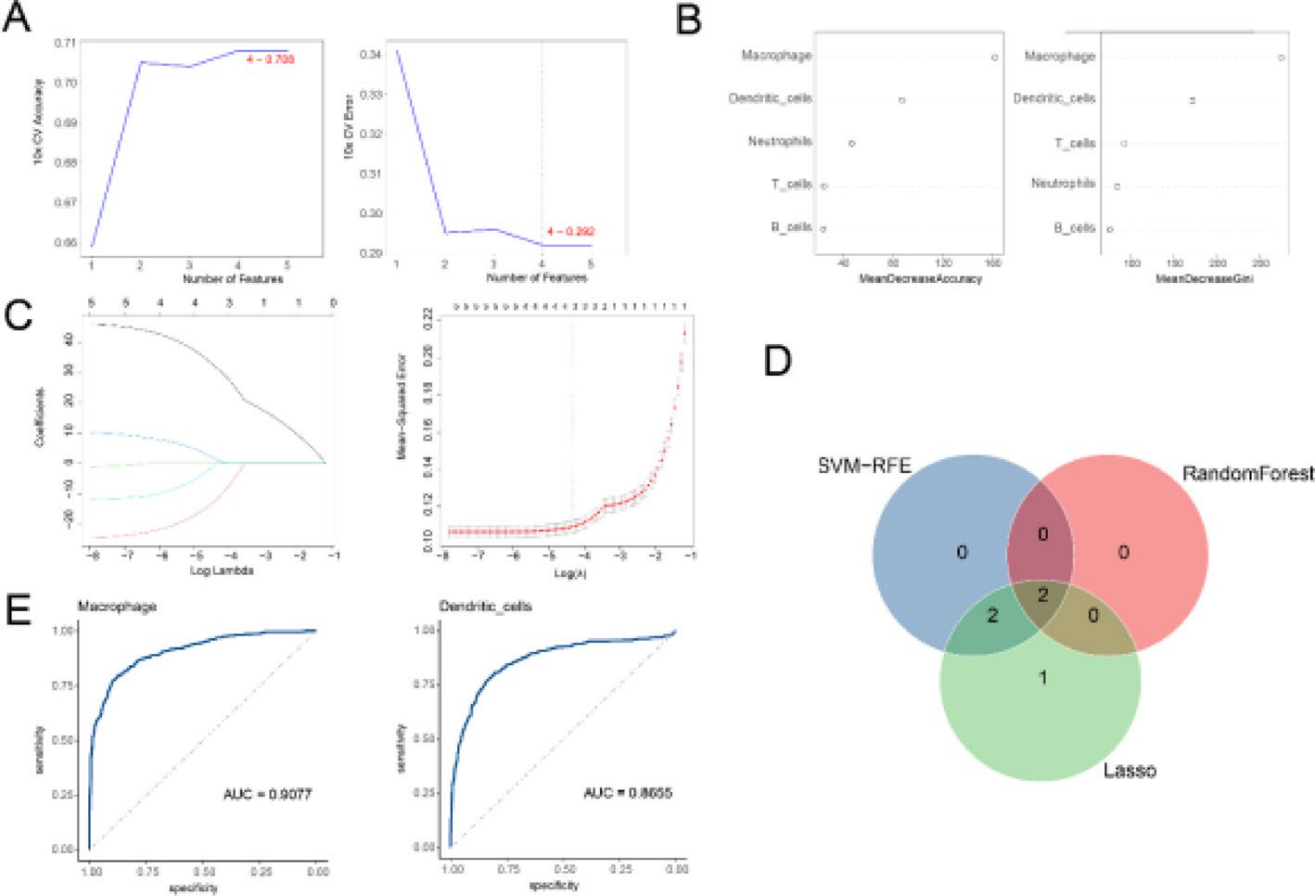
Identification of key immune cells through machine learning and evaluation of their diagnostic value.** A** SVM-RFE accuracy vs. error rate. **B** Feature importance of immune cell types from the Random Forest model. **C** Coefficient profiles for each cell type in the LASSO regression model and cross-validation error. **D** Venn diagram identifying the intersection of key features (macrophages and dendritic cells) from all three algorithms. **E** ROC curves demonstrating the strong diagnostic performance of macrophage and dendritic cell abundance for distinguishing GBM from normal samples

Using the abundance of five immune cell types (macrophages, neutrophils, T cells, dendritic cells, and B cells) derived from bulk transcriptomic data, we applied three machine learning algorithms to identify those most associated with GBM progression. The SVM-RFE algorithm shortlisted four key cell types: B cells, dendritic cells, macrophages, and neutrophils (Fig. 4A). Conversely, the LASSO regression model retained all five cell types as relevant to GBM status (Fig. 4C). For the Random Forest algorithm, it pinpointed macrophages and dendritic cells as the two most important features (Fig. 4B). Taking the intersection of the results from all three methods yielded two key immune cell types: macrophages and dendritic cells (Fig. 4D). To evaluate their diagnostic potential, we performed ROC curve analysis. This revealed strong diagnostic performance for both cell types, with macrophages achieving a particularly high AUC of 0.9 (Fig. 4E). Given their consistent selection across all algorithms and superior diagnostic performance, macrophages were identified as the most pivotal immune cell type in GBM and were therefore selected for all subsequent analyses.

### Analysis of immune responses in macrophages

**Fig. 5:**
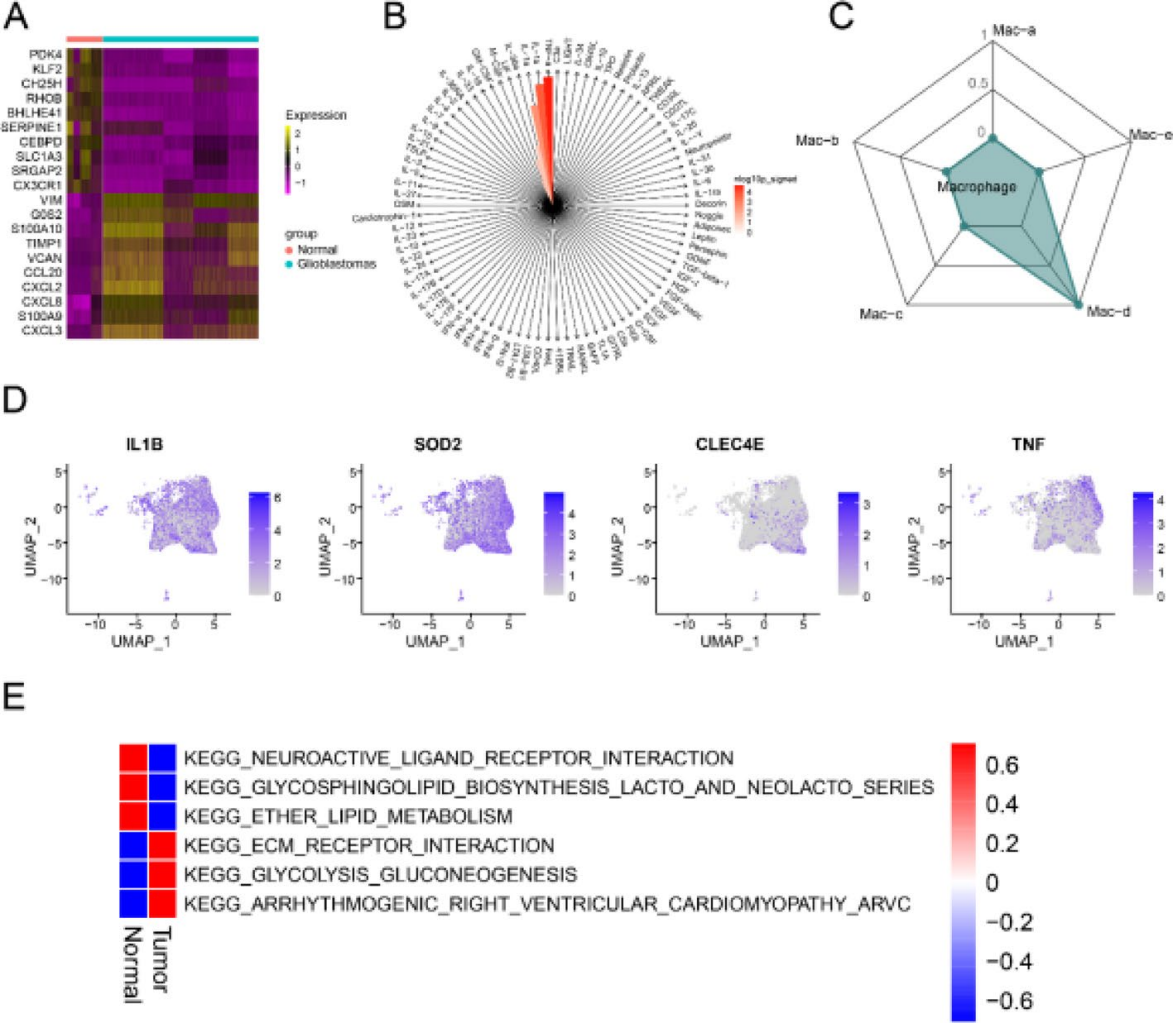
Immune landscape and functional heterogeneity of macrophages in GBM. **A** Heatmap of the top 20 differentially expressed genes in macrophages from GBM vs. normal samples. **B** IREA cytokine enrichment map showing significant activation of TNF-α signaling in GBM-associated macrophages. **C** Radar plot comparing macrophage polarization states, highlighting the dominant Mac-d (TNF-α-induced) state in GBM. **D** Violin plots (or Dot plots) showing consistent expression of Mac-d marker genes (*IL1B*,* SOD2*,* CLEC4E*,* TNF*) across macrophage samples. **E** GSVA enrichment heatmap revealing distinct functional pathways enriched in different macrophage subgroups

To investigate the immune status of macrophages in GBM, we conducted a differential expression analysis between GBM patient groups and normal control groups (e.g., healthy individuals or patients with non-tumor brain conditions; Table S6). A heatmap revealed 20 genes that were significantly differentially expressed in GBM compared to normal tissues (Fig. 5A). Using the significantly up-regulated genes in GBM, we performed IREA to assess alterations in cytokine activity following GBM development. The cytokine enrichment map indicated strong macrophage enrichment for cytokines including TNF-α in GBM (Fig. 5B). Furthermore, a polarization radar chart illustrated that macrophages in GBM predominantly exhibited a TNF-α-induced polarization state (Mac-d), which may promote inflammatory responses within the tumor microenvironment (Fig. 5C). We also examined the expression of Mac-d marker genes (*IL1B*,* SOD2*,* CLEC4E*,* TNF*) in macrophages and observed consistent expression across samples (Fig. 5D) [24], supporting the pro-inflammatory role of macrophages during gliomagenesis. To evaluate functional heterogeneity among macrophage subgroups, we performed Gene Set Variation Analysis (GSVA) enrichment analysis, which revealed distinct functional profiles between groups (Fig. 5E).

### Macrophage reclustering

**Fig. 6:**
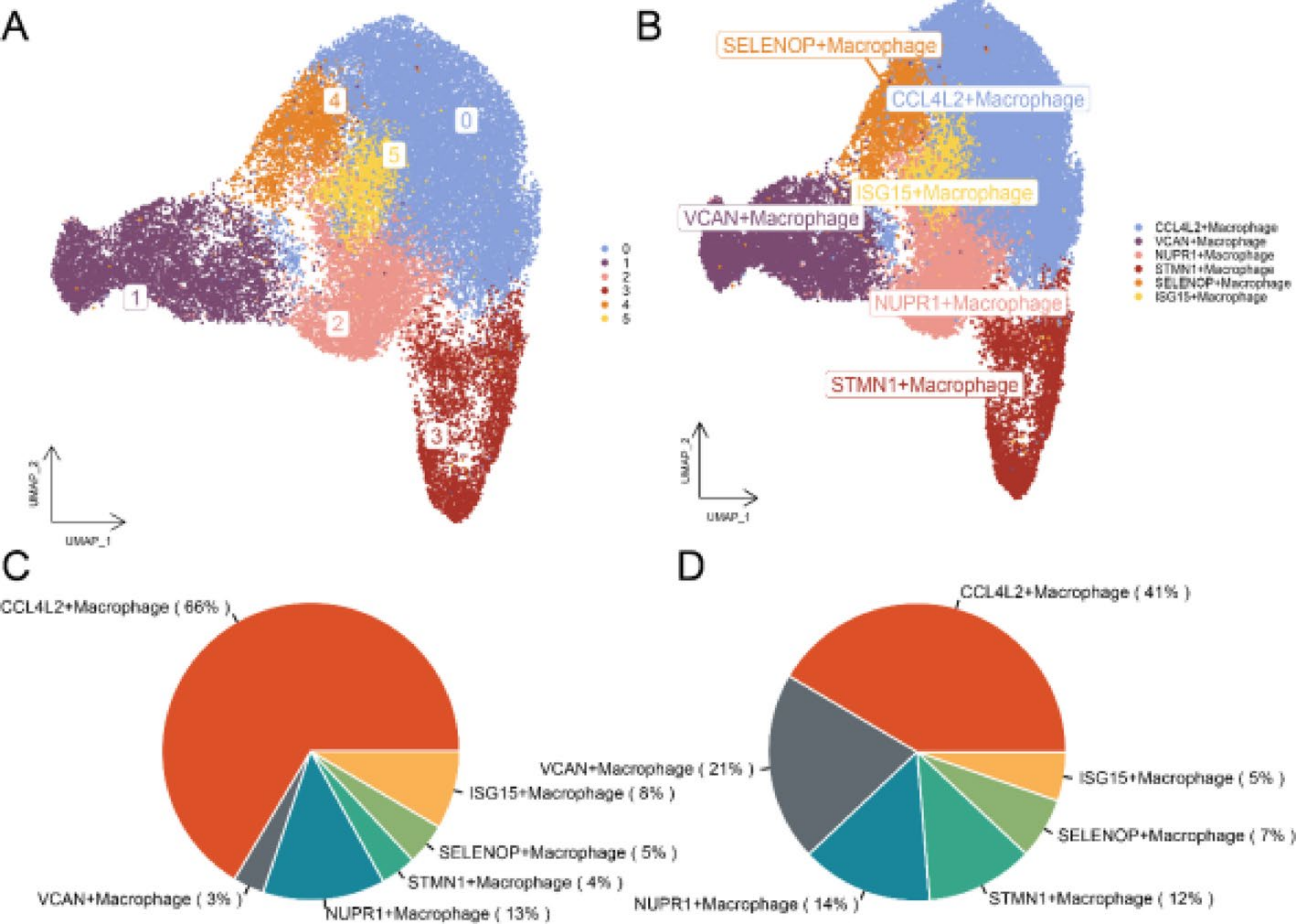
Heterogeneity and proportional shifts of macrophage subpopulations in GBM. **A** UMAP visualization of six macrophage subclusters derived from reclustering analysis. **B** UMAP plot annotated by macrophage subcluster identity. **C**, **D** Pie charts showing the proportional distribution of macrophage subclusters in normal (**C**) and GBM tumor (**D**) samples

To elucidate macrophage heterogeneity, we performed unsupervised secondary reclustering analysis on the previously annotated macrophage population (extracted from the global 35 clusters and 9 major cell type annotation, Fig. 2B-C), which identified six distinct macrophage subpopulations (Fig. 6A). These subclusters were annotated and defined purely based on their unique characteristic marker gene expression profiles and transcriptomic signatures, rather than sample group abundance (Fig. 6B). After defining each macrophage subcluster via molecular features, the proportional distribution of each subcluster across normal and tumor samples was visualized in pie charts (Fig. 6C, D). Strikingly, we observed the most pronounced shifts in the abundances of two subtypes: the tumor group exhibited a decreased proportion of *CCL4L2⁺* macrophages and a substantial increase in VCAN⁺ macrophages. These findings strongly implicate both subtypes in gliomagenesis, with VCAN⁺ macrophages likely playing a key role in GBM development.

### Macrophage pseudotime trajectory analysis

**Fig. 7:**
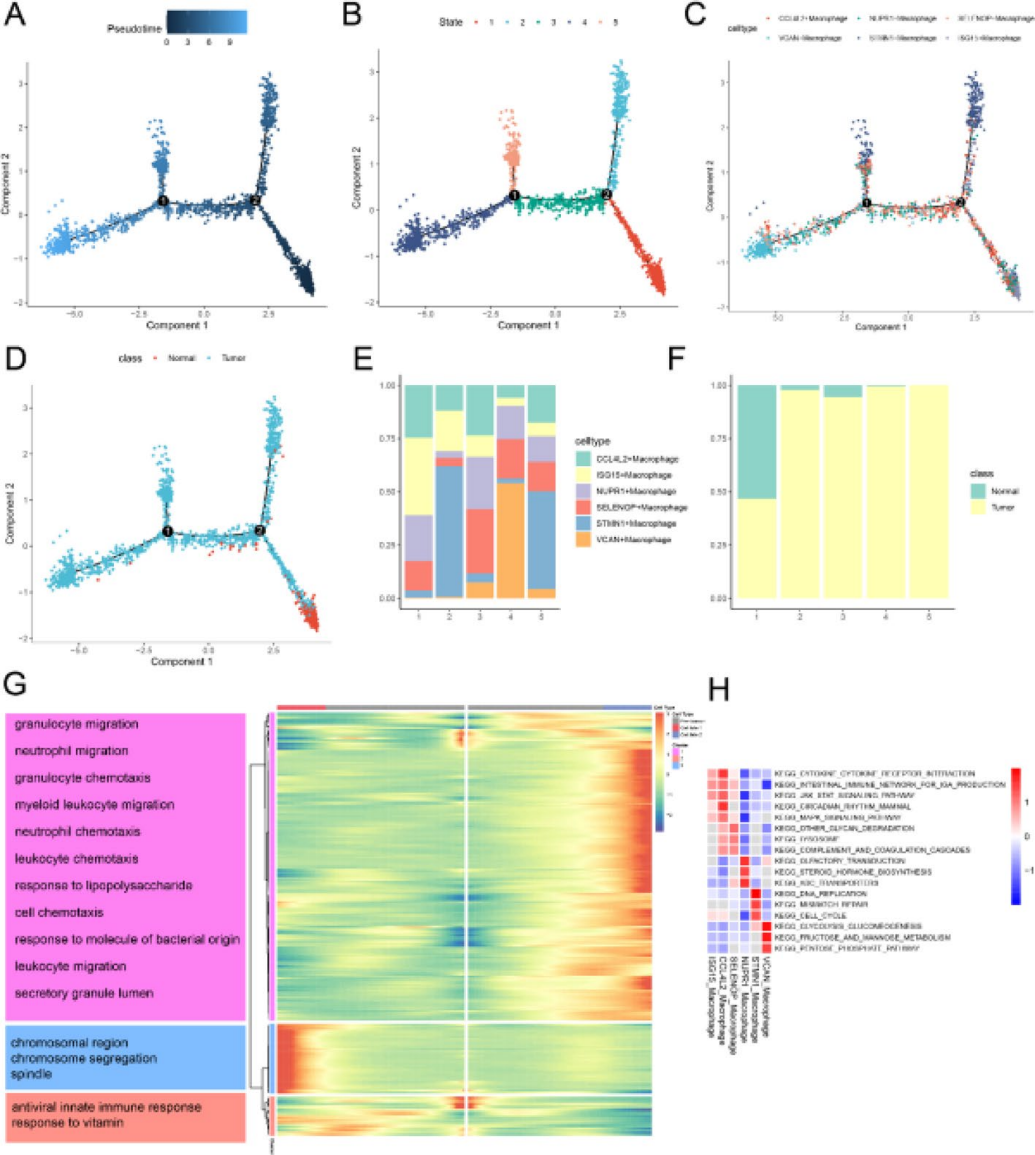
Pseudotime trajectory analysis reveals VCAN⁺ macrophages as a terminal, pro-tumoral subtype.** A** Pseudotime trajectory colored by pseudotime value (dark to light blue). **B** Cell states identified along the trajectory. **C**, **D** Trajectory colored by macrophage subtype (C) and sample group (**D**). **E** Stacked bar plot showing the proportion of macrophage subtypes in each state. **F** Stacked bar plot showing the proportion of sample groups (Normal vs. Tumor) in each state. **G** Heatmap of gene expression patterns for branching-dependent genes, with key enriched GO terms indicated. **H** Heatmap of GSVA pathway enrichment scores across macrophage subpopulations

We reconstructed a pseudotime trajectory from the macrophage subpopulations to delineate gene expression programs underlying GBM progression. This analysis revealed distinct transcriptional states mapping to dynamic biological processes. The trajectory origin (pseudotime = 0) was designated to *CCL4L2⁺*, *ISG15⁺*, and *NUPR1⁺* macrophages based on a comprehensive integration of multiple lines of evidence: first, these subpopulations exhibited a significantly higher proportion in normal samples compared to tumor samples (Fig. 6C, D), in sharp contrast to VCAN⁺ macrophages that were almost exclusively enriched in tumor tissues, suggesting the former represent macrophage subsets with a “normal homeostatic state” while the latter are terminally differentiated subtypes shaped by the tumor microenvironment; second, multiple initial pseudotime analyses with different biologically rational root cell setting strategies (e.g., taking the most abundant subpopulation in normal samples or cells with high expression of homeostasis-related genes as the root) consistently positioned *CCL4L2*⁺, *ISG15*⁺, and *NUPR1*⁺ macrophages at the trajectory origin, with VCAN⁺ macrophages stably localized at the terminal stage, demonstrating the robustness of this differentiation transcriptional state transitions independent of specific root cell selection; third, the biological functions enriched in these subpopulations (e.g., antiviral innate immune response and response to vitamin) are typical characteristics of tissue-resident or resting macrophages, forming a reasonable functional transition to the terminally differentiated state of tumor-associated macrophages. The trajectory origin (pseudotime = 0) was occupied by *CCL4L2*⁺, *ISG15*⁺, and *NUPR1*⁺ macrophages, while VCAN⁺ macrophages were positioned at the terminus. Other subtypes resided at intermediate transition states (Fig. 7A–D). Analysis of subtype distribution across states showed VCAN⁺ macrophages were highly enriched in state 4 (Fig. 7E). Critically, cells from normal samples primarily resided in the pre-differentiation stage (state 1), whereas tumor-derived macrophages were distributed across advanced differentiation states (states 2–5) (Fig. 7F). This stark dichotomy indicates a profound shift in macrophage differentiation within the tumor microenvironment.

We next interrogated the pathways driving VCAN⁺ macrophage transcriptional state transitions, focusing on branch node 1 (identified in the pseudotime trajectory; Fig. 7A–D). Pre-branch genes (states 1–3) were enriched for antiviral innate immune response, response to vitamin and gliogenesis. In contrast, branch 2 (state 4, containing VCAN⁺ macrophages) was markedly enriched for pathways mediating granulocyte and neutrophil migration (granulocyte migration, neutrophil migration, granulocyte chemotaxis, myeloid leukocyte migration). Branch 1 (state 5) showed enrichment for cell cycle-related terms (chromosomal region, chromosome segregation, spindle) (Fig. 7G, H).

Collectively, our trajectory analysis posits that VCAN⁺ macrophages represent a terminal, pro-tumoral subtype that may orchestrate a malignant cascade—from local signal amplification to systemic immune escape and tissue invasion—primarily through the recruitment and functional polarization of granulocytes. This mechanism represents a critical node in GBM progression.

### Cell communication analysis

**Fig. 8:**
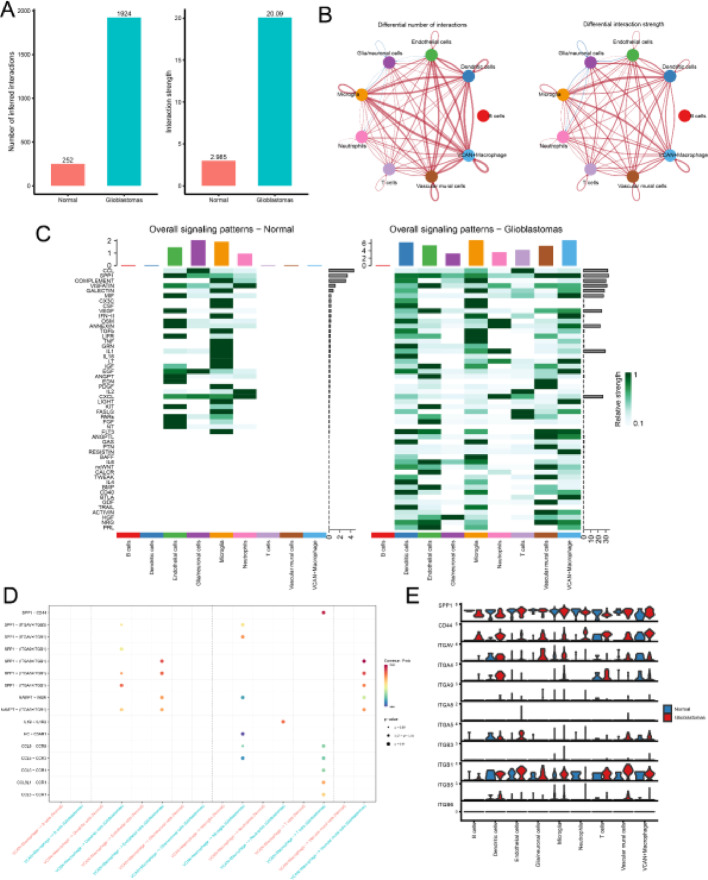
Altered intercellular communication in GBM highlights a central role for VCAN⁺ macrophages. **A** Bar graph quantifying the number and strength of interactions in normal and GBM samples. **B** Network diagrams comparing interaction numbers (left) and strengths (right) between normal and GBM. Red and blue lines indicate increases and decreases in GBM, respectively. **C** Heatmap of outgoing communication patterns for each cell type in normal and GBM. **D** Bubble plot of signaling pathways with significant changes in activity between normal and GBM. **E** Violin plots showing expression distribution of the *SPP1* ligand and its key receptors (e.g., CD44, ITGAV) across cell types in normal and GBM microenvironments

To investigate the role of VCAN⁺ macrophages in gliomagenesis, we analyzed their cellular interactions with eight other cell types using the R package CellChat. This analysis revealed extensive alterations in intercellular crosstalk between normal and glioblastoma tissues. Compared to normal tissue, glioblastoma exhibited a significant increase in both the number and strength of cellular interactions (Figs. 8A, B). This indicates a more active communication network in the GBM microenvironment, wherein VCAN⁺ macrophages participate extensively as a major signaling source.

We next compared signaling patterns with a specific focus on VCAN⁺ macrophages. The overall patterns show that this subset exhibits enhanced outgoing signaling in glioblastoma (Fig. 8C). In particular, SPP1 signaling from VCAN⁺ macrophages to multiple recipient cell types were markedly strengthened (Fig. 8D). To elucidate the mechanistic basis, we examined the expression of relevant ligand–receptor pairs. Violin plots revealed distinct expression distributions of the SPP1 ligand and integrin-related receptors—such as *CD44* and *ITGAV*—between normal and glioblastoma microenvironments. Notably, VCAN⁺ macrophages in glioblastoma expressed higher levels of SPP1, accounting for the increased activity in SPP1-associated pathways.

This implies that VCAN⁺ macrophages not only support granulocyte recruitment but also play an important role in the cellular communication network of glioblastoma, linking immunosuppression, vascular remodeling, and stromal degradation.

### Single-cell transcription factor analysis

**Fig. 9:**
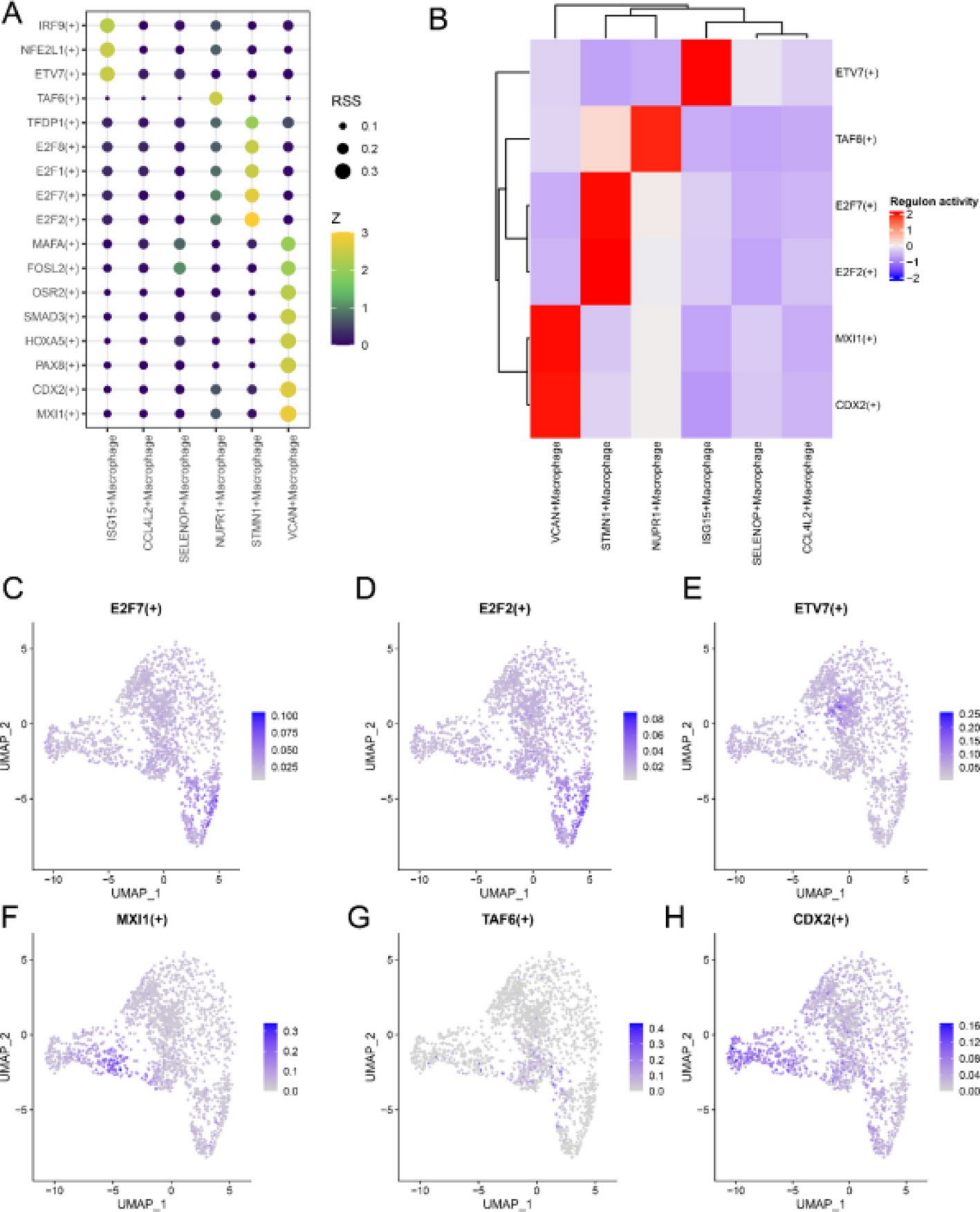
VCAN⁺ macrophages are defined by a core transcriptional regulatory network involving CDX2 and MXI1. **A** Dot plot of transcription factor regulons enriched across different cell types. **B** Heatmap of regulon activity across macrophage subpopulations, highlighting the specificity of CDX2 and MXI1 to VCAN⁺ macrophages. **C**, **H** Feature plots (or Violin plots) visualizing the activity or expression of key transcription factors across single cells

To decipher the transcriptional regulation of macrophage phenotypes, we performed Single-Cell Regulatory Network Inference and Clustering (SCENIC) analysis to identify key regulons (sets of target genes controlled by a single transcription factor). We found that macrophage subpopulations are driven by distinct repertoires of transcription factors (TFs). Specifically, VCAN⁺ macrophages were uniquely associated with the *CDX2(+)* and *MXI1(+)* regulons (Fig. 9A–B). The activity of these TFs was highly enriched specifically in the VCAN⁺ macrophage subset (Fig. 9C-H). Functionally, we posit that *CDX2* directs an outward-facing program of recruitment and anchoring, while *MXI1* maintains an internal state of low proliferation and long-term residency. Together, *CDX2* and *MXI1* coordinate a pro-tumorigenic program that promotes granulocyte chemotaxis and sustains metabolic-immune homeostasis, enabling both invasion and survival within the GBM microenvironment.

### Screening of glioblastoma-related genes in a key macrophage subpopulation

**Fig. 10:**
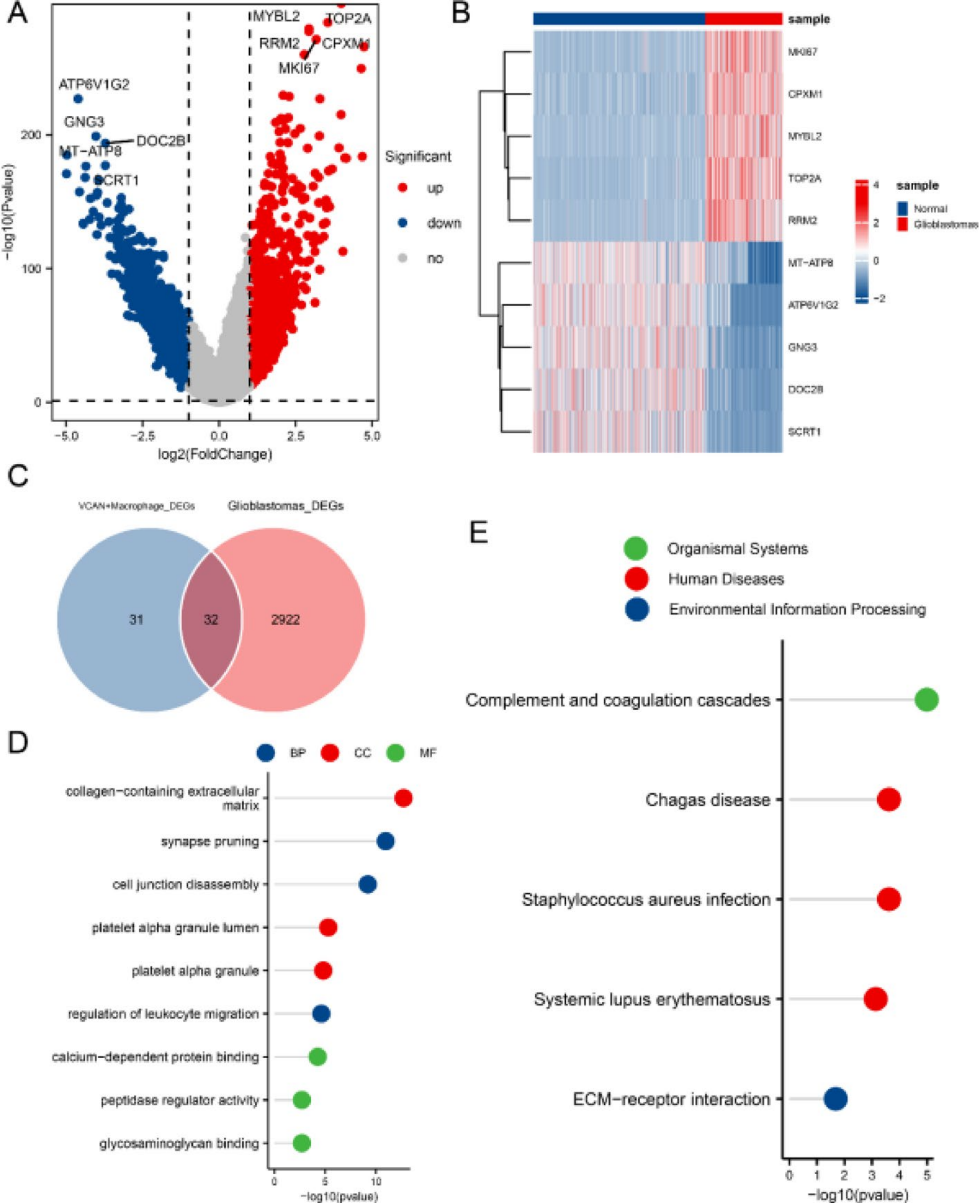
Identification of a VCAN⁺ macrophage-specific gene signature with prognostic and functional relevance in GBM.** A** Volcano plot of differentially expressed genes (DEGs) between GBM and normal samples. **B** Heatmap of the top 10 most significantly up- and down-regulated DEGs from (**A**). **C** Venn diagram identifying the intersection of DEGs from bulk tissue and VCAN⁺ macrophage-specific DEGs, yielding 32 core genes. **D**, **E** Bubble plots of the top significantly enriched GO terms (**D**) and KEGG pathways (**E**) for the 32 intersected genes

Given the central role of VCAN⁺ macrophages in GBM progression, we sought to identify key molecular drivers within this subset using bulk transcriptomic data. Differential expression analysis between GBM and normal samples identified 2954 DEGs (1,317 up; 1,637 down; |log₂ Fold Change| > 1, *p* < 0.05; Fig. 10A, Table S7). A heatmap of the top 10 most significant DEGs showed upregulation of *MKI67*,* CPXM1*,* MYBL2*,* TOP2A*, and *RRM2* and downregulation of *MT-ATP8*,* ATP6V1G2*,* GNG3*,* DOC2B*, and *SCRT1* in GBM (Fig. 10B).

To refine our focus to genes specific to the VCAN⁺ phenotype, we compared VCAN⁺ macrophages to other macrophage subgroups, identifying 63 DEGs (|log₂ Fold Change| > 1.5, *p* < 0.05; Table S8). The intersection of these two gene sets pinpointed 32 high-confidence, VCAN⁺ macrophage-specific genes linked to GBM (Table S9, Fig. 10C).

GO and KEGG enrichment analyses of these 32 key genes (identified from VCAN⁺ macrophage-specific differentially expressed genes/regulon target genes) revealed their multifaceted roles (Tables S10, S11). GO terms were significantly enriched for processes including synapse pruning, leukocyte migration, and cell junction disassembly; cellular components such as the collagen-containing extracellular matrix; and functions like calcium-dependent protein binding (Fig. 10D). KEGG analysis highlighted involvement in ECM–receptor interaction, complement and coagulation cascades, and Staphylococcus aureus infection (Fig. 10E).

Collectively, this functional profile suggests that the 32-gene signature is not only a marker of VCAN⁺ macrophages but also encodes a functional program critical for extracellular matrix remodeling, immune cell recruitment, and modulation of the tumor microenvironment—processes essential for GBM pathogenesis.

### Screening of hub genes through machine learning

**Fig. 11:**
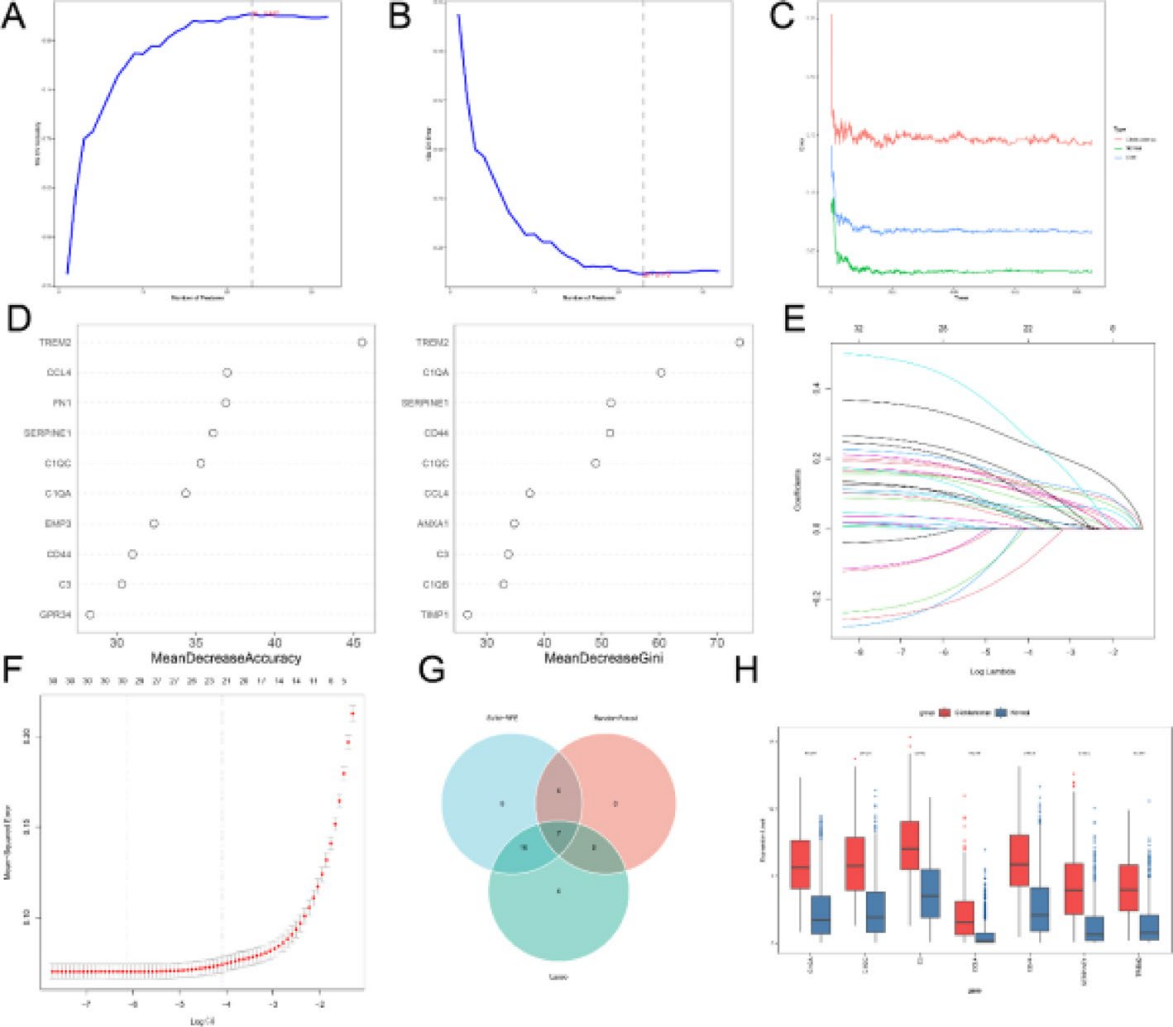
Robust identification of hub genes via integrative machine learning. **A**,** B** SVM-RFE algorithm performance: number of features vs. error rate (**A**) and 10-fold cross-validation error for optimal feature selection (**B**). **C** Random forest model error rate vs. number of trees. **D** Top 10 most important genes from the random forest algorithm based on mean decrease accuracy (MDA) and mean decrease Gini (MDG). **E** LASSO coefficient profiles for all genes. **F** LASSO cross-validation curve showing deviance vs. log(λ). **G** Venn diagram of the gene sets identified by SVM-RFE, random forest (RF), and LASSO, highlighting the seven-gene intersection. **H** Box plots demonstrating differential expression of the seven validated hub genes between normal and glioblastoma samples

To distill the most central regulators from the 32 key genes, we employed a robust machine learning approach integrating three distinct algorithms. The SVM-RFE algorithm identified 23 key genes (Figs. 11A-B). The random forest algorithm, based on both MDA and MDG metrics, converged on a set of seven key genes (Figs. 11C-D). LASSO regression analysis selected 29 key genes (Figs. 11E-F). Taking the intersection of the results from all three methods yielded a high-confidence core of seven hub genes: *C1QA*,* C1QC*,* C3*,* CCL4*,* CD44*,* SERPINE1*, and *TREM2* (Fig. 11G). Box plots confirmed that all seven hub genes were significantly differentially expressed between normal and glioblastoma samples (Fig. 11H).

This multi-algorithmic approach robustly identified a compact gene signature deeply associated with glioblastoma, encompassing critical players in complement signaling (*C1QA*,* C1QC*,* C3*), chemotaxis (*CCL4*), extracellular matrix interaction (*CD44*), coagulation (*SERPINE1*), and myeloid cell activation (*TREM2*).

### Prognostic model based on hub genes

**Fig. 12:**
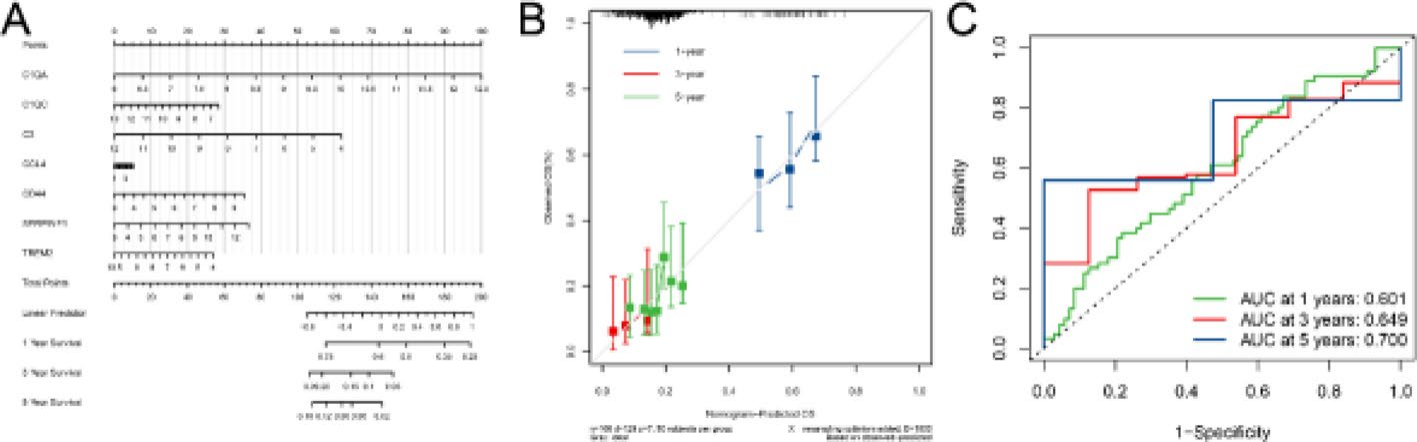
A prognostic nomogram model based on hub genes and its validation. **A** The prognostic nomogram. Each variable is assigned a score on the top point scale. The sum of all scores corresponds to a probability of 1-, 3-, and 5-year overall survival on the bottom scales. **B** Calibration plots of the nomogram for predicting 1-, 3-, and 5-year survival. The 45-degree dotted line represents perfect prediction. **C** Time-dependent receiver operating characteristic (ROC) curves demonstrating the discriminatory ability of the nomogram at 1-, 3-, and 5-year. AUC values are indicated

To assess the clinical prognostic value of the seven hub genes, we first identified genes with independent prognostic value for patient survival via univariate and multivariate Cox proportional hazards regression analyses; we then constructed a prognostic nomogram incorporating these independently predictive genes (all derived from the seven hub genes; Fig. 12A).The model demonstrated high predictive accuracy, as evidenced by the calibration curves, which showed excellent agreement between predicted and observed 1-, 3-, and 5-year survival probabilities (Fig. 12B). Furthermore, time-dependent ROC analysis confirmed the model’s strong discriminatory power, with high Area Under the Curve (AUC) values at each time point (Fig. 12C).

## Discussion

Using machine learning approaches applied to single-cell RNA sequencing data, we identified macrophages as the most critical immune cell type in the GBM microenvironment. These macrophages exhibited substantial heterogeneity, with the VCAN⁺ macrophage subpopulation being particularly enriched in tumor samples. VCAN is a large chondroitin sulfate proteoglycan that contributes to extracellular matrix organization and participates in various pathological processes. It is aberrantly expressed in multiple cancers, where it promotes tumor invasion and progression by modulating cell adhesion, proliferation, migration, and angiogenesis [32, 33]. VCAN also regulates tumor cell behavior through interactions with cell surface receptors such as *CD44* and integrins [34]. Importantly, VCAN influences not only tumor cells but also the tumor immune microenvironment. It can affect macrophage polarization via Toll-like receptor 2 (*TLR2*), and its proteolytic fragment versikine can promote the production of pro-inflammatory cytokines including IL-1β and IL-6, thereby shaping the immune landscape of tumors [35]. Our study identifies a distinct VCAN⁺ macrophage subset within the GBM microenvironment. This discovery extends the current understanding of TAM heterogeneity and function in GBM, providing a strong rationale for developing novel immunotherapeutic strategies that specifically target this pro-tumoral population.

Based on pseudotime analysis, VCAN⁺ macrophages occupied a terminal position in the differentiation trajectory and were predominantly localized in State 4, suggesting they represent a distinct, late-differentiation subpopulation. *CCL4L2+*, *ISG15+*, and *NUPR1 +* macrophages were positioned near the trajectory origin, with other subtypes distributed along intermediate transitional phases [36]. A critical branching point (Node 1) marked the commitment to the VCAN⁺ macrophage transcriptional state transitions, indicating a decisive shift from normal to a tumor-associated state. Pathway analysis of this branch revealed significant enrichment for granulocyte and neutrophil migration and chemotaxis in State 4 [37], highlighting a primary role in recruiting myeloid cells to the tumor niche. This enrichment implies that VCAN⁺ macrophages may orchestrate a pro-tumoral cascade—from local signal amplification to systemic immune escape and ultimately tissue invasion—via multi-pathway-mediated granulocyte recruitment and functional polarization [38]. Accumulating recent studies have demonstrated that the classical M1/M2 binary classification system is inadequate to fully depict the authentic functional states of tumor-associated macrophages (TAMs) in glioblastoma and other solid tumors, as TAMs frequently exhibit hybrid or dynamically plastic phenotypic characteristics in response to the complex tumor microenvironment. Specifically, TNF-α exerts dual and context-dependent biological effects within the tumor microenvironment: on one hand, it acts as a canonical pro-inflammatory cytokine that participates in anti-tumor immune responses under physiological conditions; on the other hand, persistent TNF-α signaling can reprogram macrophages to secrete potent immunosuppressive factors including IL-10 and TGF-β, and indirectly facilitate tumor progression by promoting angiogenesis and extracellular matrix remodeling. The Mac-d polarization state identified in our study represents a unique hybrid phenotype driven by sustained TNF-α stimulation, which harbors partial pro-inflammatory signatures typified by high expression of *IL1B* and *SOD2*, alongside robust pro-tumorigenic and immunosuppressive functions mainly mediated by *SPP1*, including enhanced immune suppression and tumor cell invasion. This hybrid activation profile is highly consistent with the phenomenon of a continuous mixed polarization spectrum of TAMs reported in recent relevant studies [39–41]. Notably, VCAN⁺ macrophages display a hybrid functional state that diverges from the conventional M1/M2 dichotomy, instead adopting a unique polarization state adapted to the glioblastoma microenvironment [42]. Collectively, these findings establish VCAN⁺ macrophages as a central orchestrator of glioblastoma progression and emphasize the critical need to dissect their precise biological mechanisms [34].

Immune response enrichment analysis (IREA) revealed that macrophages in the GBM microenvironment are predominantly enriched for cytokines including TNF-α—consistent with this, VCAN⁺ macrophages exhibited a specific TNF-α–induced polarization state (Mac-d), characterized by high expression of activation markers such as *IL1B*, *SOD2*, *CLEC4E*, and *TNF* [24]. This state supports a pronounced pro-inflammatory role for VCAN⁺ macrophages within the GBM niche, yet it reflects a more complex functional spectrum that cannot be simply categorized within the conventional M1/M2 dichotomy. VCAN, as a key extracellular matrix protein, is known to regulate immune responses across various cancers. For instance, in colorectal cancer, VCAN processing regulates the immune microenvironment and correlates with CD8⁺ T-cell infiltration; its degradation product promotes the differentiation of specific dendritic cell subsets [43].

In GBM, our data suggest that VCAN⁺ macrophages are activated via TNF-α signaling and may subsequently establish a pro-tumorigenic niche. The association of high VCAN expression with poor prognosis in multiple cancers [42] aligns perfectly with our functional assignment of a tumor-promoting role to VCAN⁺ macrophages. Thus, our findings not only provide new insights into the complexity of macrophage polarization in GBM but also challenge the constraints of the traditional binary classification. Moreover, they strongly support the therapeutic feasibility of targeting VCAN⁺ macrophages to disrupt this key node of tumor progression.

Cell communication analysis revealed a substantial increase in both the number and strength of interactions within the GBM microenvironment. Notably, VCAN⁺ macrophages occupied a central position within this hyperconnected network. A key mediator of this communication was SPP1 (osteopontin) signaling from VCAN⁺ macrophages, which was markedly enhanced in GBM and targeted multiple recipient cell types. The cognate receptors for *SPP1*—including *CD44* and *ITGAV*—were critically involved in mediating these interactions. Violin plots confirmed that VCAN⁺ macrophages are the primary source of elevated *SPP1* expression in GBM, accounting for the enhanced activity of *SPP1*-associated pathways [27]. This establishes a reprogrammed communication network wherein VCAN⁺ macrophages, via *SPP1* secretion, activate granulocyte recruitment pathways and position themselves at the core of a pro-tumorigenic ecosystem. Functionally, *SPP1* promotes tumor migration, invasion, and angiogenesis by binding to *CD44* and integrin receptors [33]. Thus, through the *SPP1* signaling axis, VCAN⁺ macrophages functionally integrate three key hallmarks of cancer progression: immunosuppression (via recruiting immunosuppressive granulocytes that suppress anti-tumor T-cell activity), vascular remodeling (via promoting angiogenesis), and matrix degradation (via enhancing tumor cell invasion). This establishes them as a critical signaling hub within the GBM microenvironment—a role consistent with the broader function of VCAN in promoting adhesion, proliferation, migration, and angiogenesis across tumor types [32, 44]. These findings provide a mechanistic basis for the pivotal role of VCAN⁺ macrophages in GBM progression, defining them as central regulators of the tumor communicative landscape.

Furthermore, SCENIC analysis identified *CDX2* and *MXI1* as key transcription factors regulating the phenotype of VCAN⁺ macrophages. We propose a functional dichotomy: *CDX2* primarily orchestrates external functions such as recruitment and anchoring, while *MXI1* maintains an internal state of homeostasis characterized by low proliferation and long-term residency. *MXI1*, a critical component of the Myc–Max–Mad network, represses c-Myc-driven gene expression by promoting chromatin condensation via histone deacetylase and the Sin3 co-repressor [45]. Its role in sustaining the quiescent state of VCAN⁺ macrophages is further supported by its ability to inhibit proliferation via regulating IGFBP-3 expression; indeed, MXI1-deficient mice exhibit aberrant proliferation and tumorigenesis [46]. The regulatory landscape of *MXI1* is complex: an alternative isoform, MXI1-0, shares DNA-binding domains but localizes to the cytoplasm and does not suppress c-Myc [45], instead modulating cell growth through IL-8 and ERK1/2 signaling [47]. Moreover, *MXI1* expression is directly regulated by HIF proteins, linking it to the hypoxic tumor microenvironment [48], and is subject to post-translational regulation via the mTOR/S6K1 pathway, wherein phosphorylation triggers its ubiquitination and degradation affecting Myc activity [49]. The synergistic action of *CDX2* and *MXI1* enables VCAN⁺ macrophages to coordinately execute a pro-tumoral transcriptional program that promotes granulocyte chemotaxis and sustains an immune-suppressive homeostasis (coupled with metabolic adaptation to the hypoxic GBM microenvironment). In contrast to previous reports in other cellular contexts, our findings uncover the specific and coordinated roles of *CDX2* and *MXI1* in defining the VCAN⁺ macrophage phenotype, offering novel insights into the maintenance of the immunosuppressive microenvironment in GBM.

Employing a machine learning approach, we identified seven hub genes (*C1QA*, *C1QC*, *C3*, *CCL4*, *CD44*, *SERPINE1*, and *TREM2*) from VCAN⁺ macrophage signature genes, each playing a pivotal role in GBM pathogenesis. These hub genes form a cooperative network driving tumor progression: Complement components *C1QA*,* C1QC*, and *C3* modulate immune-inflammatory responses and promote an immunosuppressive microenvironment, with *C1QA/C1QC* expression linked to macrophage polarization states [50, 51]. *TREM2*, a macrophage surface receptor, enhances oxidative stress and regulates complement expression and phagocytosis, thereby exacerbating neuroinflammation [50, 52]. *CCL4* and *CD44* contribute to cellular chemotaxis and extracellular matrix reorganization. *CD44* also acts as a key receptor for *SPP1*, mediating critical intercellular communication [53]. *SERPINE1* participates in coagulation cascades and regulates extracellular matrix degradation—processes directly associated with enhanced tumor invasiveness [54]. A prognostic model constructed from these hub genes demonstrated high predictive accuracy and robustness. Calibration and ROC curve analyses confirmed its utility in predicting 1-, 3-, and 5-year survival in GBM patients. Collectively, this hub gene signature encapsulates a functional network spanning immunosuppression to matrix remodeling, offering a compelling rationale for targeting VCAN⁺ macrophages immunologically [55]. “Notably, existing strategies targeting *TREM2 +* macrophages have shown potential to enhance immunotherapeutic responses [50]. Given that our identified hub genes are specific to VCAN⁺ macrophages and correlate with patient survival, they could inform novel avenues for personalized GBM therapy—for example, using the 7-gene signature to stratify patients likely to benefit from VCAN⁺ macrophage-targeted treatments, or developing combination therapies targeting *TREM2* and complement components (*C1QA/C1QC/C3*) to disrupt the pro-tumoral network of VCAN⁺ macrophages.” Future studies will focus on validating the universality of the VCAN⁺ macrophage subpopulation in larger, multi-center GBM scRNA-seq cohorts. Specifically, we aim to integrate independent datasets that include paired tumor and normal samples, sufficient sample sizes, and comparable sequencing platforms and cell annotation pipelines. Cross-dataset validation will help confirm the conserved presence and functional characteristics of VCAN⁺ macrophages across diverse patient populations, thereby strengthening the translational potential of this subpopulation as a therapeutic target and prognostic biomarker in GBM.

## Limitations

Although this study highlights the important role of VCAN⁺ macrophages in the glioblastoma (GBM) microenvironment, several limitations should be acknowledged. First, this study relied on a single scRNA-seq dataset (GSE162631) comprising only four paired tumor core and peritumoral samples. Although the paired design enabled direct comparison between tumor and adjacent tissues, and the dataset provided relatively high sequencing depth and cell quality, the limited sample size may have reduced the statistical power to capture the full heterogeneity of the GBM microenvironment. It also raises the possibility that the identified VCAN⁺ macrophage subpopulation may be, at least in part, dataset-specific.

Second, the GSE162631 dataset was generated following magnetic-activated cell sorting for enrichment of *CD31⁺* GBM endothelial cells. This enrichment strategy likely impaired the effective capture of *CD31⁻* cell populations, particularly neurons and astrocytes, resulting in their underrepresentation in the original dataset and their exclusion from subsequent annotation and analysis. As a consequence, our study may not fully reflect the complete cellular composition of the GBM microenvironment. In addition, accurate annotation of neurons and astrocytes remains intrinsically challenging because of the substantial transcriptomic overlap among neurons, reactive astrocytes, and tumor cells, including the shared expression of several marker genes.

Third, although our bioinformatics analyses identified key transcriptional programs, intercellular communication pathways, and prognostically relevant hub genes associated with VCAN⁺ macrophages, these findings remain computationally inferred and require further experimental validation. In particular, in vitro and in vivo functional studies will be necessary to confirm the causal role of VCAN⁺ macrophages in tumor progression, including experiments examining the effects of VCAN⁺ macrophage-conditioned medium on tumor cell behavior and targeted depletion strategies in animal models.

Another limitation is that we did not explicitly resolve the lineage origin of VCAN⁺ macrophages, namely whether they derive predominantly from resident microglia or infiltrating monocyte-derived macrophages. Although these two populations differ in developmental origin, transcriptional features, and biological functions in GBM, the endothelial cell enrichment strategy used in GSE162631 resulted in partial absence or incomplete coverage of canonical lineage markers, such as *P2RY12* and *TMEM119* for microglia, and *CCR2* and *FCN1* for monocyte-derived macrophages. This may have compromised the accuracy of strict lineage classification. Moreover, emerging evidence [56] suggests that resident microglia and infiltrating macrophages may undergo substantial functional convergence and transcriptional overlap during GBM progression, particularly in terminally differentiated states, thereby further reducing the discriminatory power of classical lineage markers. Therefore, under the constraints of the available data, our study focused primarily on the functional pro-tumoral properties of VCAN⁺ macrophages rather than on defining their precise ontogeny.

Future studies should address these limitations by expanding sample size across diverse molecular subtypes of GBM, integrating independent scRNA-seq cohorts with more complete lineage-marker coverage, and applying higher-resolution approaches such as single-cell multi-omics and spatial transcriptomics. In addition, lineage-tracing strategies in preclinical models will be essential for clarifying the developmental origin and dynamic evolution of VCAN⁺ macrophages. These efforts will help refine the classification of this macrophage subpopulation, improve understanding of its spatial and functional niche within the GBM microenvironment, and further evaluate its translational and therapeutic potential.

## Conclusion

This study identifies VCAN⁺ macrophages as a pivotal immune subpopulation in the GBM microenvironment through an integrated scRNA-seq and bioinformatics. These cells exhibit a TNF-α–induced Mac-d polarization state—a unique functional spectrum distinct from the conventional M1/M2 dichotomy—that promotes tumor migration, invasion, and immune escape primarily via the *SPP1* signaling pathway. Pseudotime trajectory analysis positioned VCAN⁺ macrophages at a terminal differentiation branch enriched for granulocyte migration and chemotaxis pathways, highlighting their multifaceted pro-tumor role. The transcriptional regulators *CDX2* and *MXI1* were identified as core nodes sustaining this subpopulation’s homeostasis, balancing “recruitment and anchoring” functions with low proliferation and long-term residency. From this, we derived a robust seven-hub-gene signature (*C1QA*, *C1QC*, *C3*, *CCL4*, *CD44*, *SERPINE1*, and *TREM2*) that participates in immune regulation and extracellular matrix reorganization. This signature not only elucidates a key molecular network but also forms the basis of a prognostic model for individualized risk stratification in GBM patients.

Our findings fundamentally enhance the understanding of the GBM immune microenvironment complexity and expand knowledge of macrophage polarization diversity in glioblastoma.These insights reveal novel immunotherapeutic targets, such as the *SPP1* pathway and *TREM2 +* macrophages, paving the way for precision treatment strategies. The hub gene-based prognostic model holds direct clinical translatability for improving risk stratification and therapeutic decision-making. We envision that future studies—incorporating larger cohorts, functional validation in vitro and in vivo, and spatial transcriptomics—will elucidate the spatial dynamics of VCAN⁺ macrophages and ultimately advance targeted therapies for this aggressive malignancy.

## Supplementary Material

Supplementary Material 1. Figure S1. Immunohistochemical validation of hub gene expression in GBM tissues from the Human Protein Atlas (HPA) database. Representative immunohistochemistry images showing the protein expression patterns of the seven hub genes in GBM tissues: (A) C1QA, (B) C1QC, (C) C3, (D) CCL4, (E) CD44, (F) SERPINE1, and (G) TREM2. Among these proteins, CD44 showed strong staining in high-grade GBM tissue, whereas C1QC, C3, and TREM2 exhibited moderate expression. C1QA and CCL4 showed relatively weak staining, and SERPINE1 was not detected in the representative GBM sample. Clinical and staining information for each specimen, including age, sex, tissue location, GBM grade, staining intensity, and quantity, are shown alongside the corresponding images.

## Data Availability

The datasets used and/or analyzed during the current study are publicly available from the following official repositories:1.GSE162631: https://www.ncbi.nlm.nih.gov/gds/?term=GSE1626312.Chinese Glioma Genome Atlas (CGGA): http://www.cgga.org.cn/index.jsp. The glioma mRNA sequencing datasets PRJCA001747 (*n*=693, https://ngdc.cncb.ac.cn/bioproject/browse/PRJCA001747) and PRJCA001746 (*n*=325, https://ngdc.cncb.ac.cn/bioproject/browse/PRJCA001746) were downloaded and screened for this study.3.The Cancer Genome Atlas (TCGA)-GBM: https://portal.gdc.cancer.gov/projects/TCGA-GBM. Whole-genome expression profile data in TPM format and corresponding clinical data were obtained via the R package TCGAbiolinks (version 2.25.0)0.4.UCSC Xena database: https://xenabrowser.net/datapages/. Integrated expression matrices of TCGA and Genotype-Tissue Expression (GTEx) data were downloaded for differential expression analysis between glioma and normal brain tissues.All analytical codes used in the current study are available from the corresponding authors on reasonable request.

## Abbreviations

GBM: Glioblastoma
TME: Tumor micro environment
TAMs: Tumor-associated macrophages TAMs
scRNA-seq: Single-cell RNA sequencing
TNF-α: Tumor necrosis factorα
MRI: Magnetic resonance imaging
MDMs: Marrow-derived macrophages
GEO: Gene Expression Omnibus
UMI: Unique Molecular Identifier
PCs: Principal components
SNN: Shared Nearest Neighbor
UMAP: Uniform Manifold Approximation and Projection
PCA: Principal component analysis
CGGA: Chinese glioma genome atlas
TCGA: The Cancer Genome Atlas
SNV: Single nucleotide variant
GSVA: Gene set variation analysis
GTEx: Genotypic Tissue Expression
ssGSEA: Single-sample Gene Set Enrichment Analysis
SVM-RFE: Support Vector Machine-recursive Feature Elimination
IREA: Immune Response Enrichment Analysis
LASSO: Least absolute shrinkage and selection operator
BH: Benjamini-Hochberg
BEAM: Branch expression analysis modeling
TFs: Transcription factors
DEGs: Differentially expressed genes
BP: Biological process
MF: Molecular function
ECM: Extracellular matrix
CC: Cellular component
KEGG: Kyoto Encyclopedia of Genes and Genomes
MDA: Mean decrease in accuracy
MDG: Mean decrease in gini
ROC: Receiver operating characteristic
AUC: Area under the curve
RF: Random forest
HPA: Human protein atlas
GO: Gene ontology
