# Supplementary figures and images for: Single-cell profiling identifies a pro-tumoral VCAN positive macrophage subset and defines a prognostic signature in glioblastoma
Source: Discov Oncol. 2026 May 9;17:969. doi: 10.1007/s12672-026-05166-y (PMC13323438; doi:10.1007/s12672-026-05166-y)

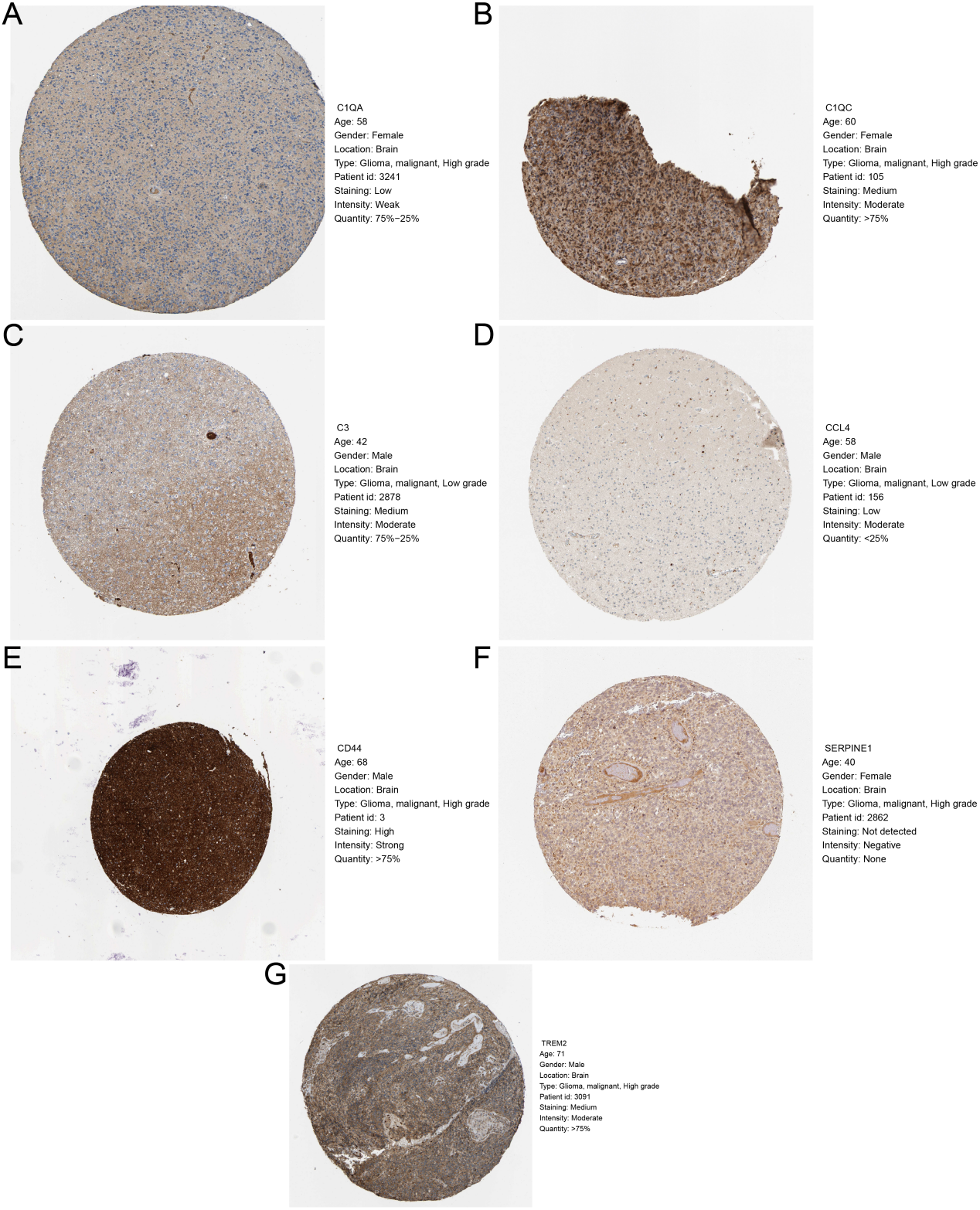

Supplement: Supplementary file 1 — Supplementary Material 1. Figure S1. Immunohistochemical validation of hub gene expression in GBM tissues from the Human Protein Atlas (HPA) database. Representative immunohistochemistry images showing the protein expression patterns of the seven hub genes in GBM tissues: (A) C1QA, (B) C1QC, (C) C3, (D) CCL4, (E) CD44, (F) SERPINE1, and (G) TREM2. Among these proteins, CD44 showed strong staining in high-grade GBM tissue, whereas C1QC, C3, and TREM2 exhibited moderate expression. C1QA and CCL4 showed relatively weak staining, and SERPINE1 was not detected in the representative GBM sample. Clinical and staining information for each specimen, including age, sex, tissue location, GBM grade, staining intensity, and quantity, are shown alongside the corresponding images. [file 12672_2026_5166_MOESM1_ESM.docx]
